# The Traveler’s Tree

**Published:** 1888-06

**Authors:** 


### THE TRAVELER'S TREE.

One of the greatest wonders of Madagascar, so famous for its luxuriant vegetation, is the traveler's tree (Ravenala Madagascariensis). Its stem resembles that of the plantain, with which it is otherwise allied ; but it sends out its wing-like leaves only on two opposite sides, which resemble a large expanded fan. In an aged tree the lowest of these leaves will be from twenty to forty feet from the ground, and on a vigorous trunk there will generally be, at least, a score of them with a bright emerald green oblong blade from four to six feet in length.

The fruit grows in bunches, containing forty or fifty members, with three or four such bunches to a tree. Each fruit member contains a quantity of the silkiest fibre imaginable, of a beautiful purple tint enclosing thirty to thirty-five seeds. The leaves are used for roof thatching and the leaf stalks twirled together, serve for the walls of the islanders' huts. But the most remarkable property of this tree, and the one which gives it the distinctive appellation of Traveler's Tree, is its petioles, which, even in the dryest seasons, always contain water, and the wayfarer, if he be thirsty, has only to pierce the thick base of a leaf stock, to obtain fully a quart of a pure and refreshing liquid.

It is needless to say that this bountiful source of vivication is greatly prized by the natives of this far away island.

---

Chloroform Water in Washing out the Stomach—Prof. Bianchi recommends chloroform water for washing out the stomach, and says he has obtained wonderful results from its use. He reports seven cases of dyspepsia and chronic gastritis, in most of which dilation of the stomach existed. He had first used alkaline water (4 grms. natr. bicarb. to one litre of water) as a wash, but with no beneficial result. He then employed very dilute chloroform water, and found it to have the following advantages :

1. It produces marked diminution of pain, and lessens the intolerance of the stomach for food.

2. It reduces the dilation of the stomach, on account of the antiseptic action of the chloroform upon the abnormal amount of decomposition taking place in the contents of the stomach, and on account of the reflex action caused by the sudden administration of the water.

3. It produces no unpleasant symptoms ; at least none have been observed up to the present.—*Weekly Med. Rev.*

